# Shape Memory Epoxy Polymer (SMEP) Composite Mechanical Properties Enhanced by Introducing Graphene Oxide (GO) into the Matrix

**DOI:** 10.3390/ma12071107

**Published:** 2019-04-03

**Authors:** Zhengwei Yu, Zhenqing Wang, Hao Li, Jianxin Teng, Lidan Xu

**Affiliations:** College of Aerospace and Civil Engineering, Harbin Engineering University, Harbin 150001, China; yzw0514@hrbeu.edu.cn (Z.Y.); lihao0202@hrbeu.edu.cn (H.L.); tengjianxin123@126.com (J.T.); xulidan0501@126.com (L.X.)

**Keywords:** polymer composite, GO, thermal performance, mechanical performance, shape memory performance

## Abstract

Shape memory epoxy polymer (SMEP) composite specimens with different graphene oxide (GO) contents were manufactured to study the effects of GO mass fractions on epoxy polymer composites. While ensuring the shape memory effect of SMEP, the addition of GO also remarkably strengthened the mechanical performance of the polymers. Analyses of the epoxy polymer composites’ thermal, mechanical, and shape memory performance were conducted through carrying out dynamic mechanical analysis (DMA), thermogravimetric analysis (TGA), and static tensile, three-point bending, impact, and shape memory tests. Moreover, the tensile fracture, bending fracture, and impact fracture interfaces of epoxy resin composites were examined with scanning electron microscopy. The final test results indicated that when the GO content was 0.8 wt %, SMEP composites had good shape memory performance and optimum thermal and mechanical performance.

## 1. Introduce

**Figure 1:**
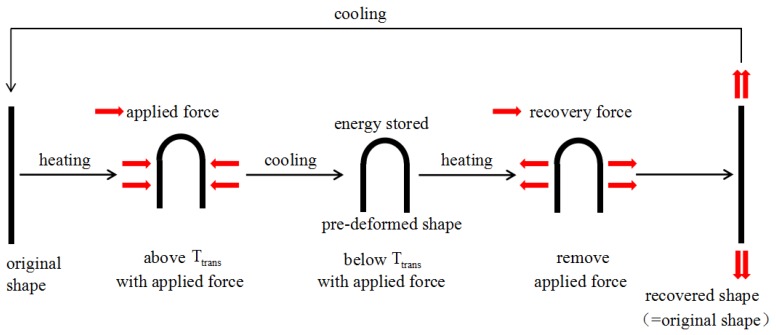
A schematic diagram of the thermal deformation process of shape memory epoxy polymer (SMEP).

Epoxy resin is an important type of thermo-responsive resin which has the advantages of good adhesion, excellent mechanical properties, abrasion resistance, and low cost. At present, it plays a very important role in the making of adhesives and in fields such as the construction industry, aerospace industry, electronics industry, and so on. Thermo-responsive epoxy resin not only has the advantages of ordinary epoxy resin, but also has good shape memory properties and can meet different needs when its chemical structure is changed [1,2,3]. Shape memory polymer composites with thermo-responsive epoxy resin as the matrix can overcome the deficits of low mechanical strength and low deformation recovery of shape memory polymers [4,5,6,7]. Shape memory epoxy polymer (SMEP) has excellent properties. It has strong shape memory, which is mainly reflected in the aspects of high shape retention, good shape recovery rate and quick response, and incomparable thermal performance and mechanical performance. Due to these properties and advantages, it has very important application prospects and a broad application space [8,9,10,11]. Figure 1 presents a schematic diagram of the thermal deformation process of SMEP. The process includes several steps: (1) heating SMEP to a temperature above the transition temperature $T_{trans}$ and subjecting it to applied force; (2) cooling SMEP until its temperature is below $T_{trans}$ and then removing the applied force; (3) reheating the pre-deformed SMEP above $T_{trans}$, at which point it will regain its original shape; and (4) cooling SMEP to a temperature below $T_{trans}$ with its original shape regained [12,13,14,15]. 

An epoxy resin system has relatively good mechanical performance, but the impact and toughness of unmodified epoxy resins are poor [16]. Furthermore, the linear expansion coefficient of epoxy resin is very large, which is likely to produce large thermal stress. Because of that, cracks and damage may be produced inside the resin, which shows greater brittleness [17,18]. This phenomenon occurs because the epoxy resin will produce large internal stress after being cured. Moreover, because the cross-linking density is very high and the structure is three-dimensionally reticulated, movement of the cured epoxy resin is difficult, and the epoxy resin’s flexibility will become poor.

Graphene is a kind of carbon and takes the allotrope form. It is made up of atoms which have two dimensions and are single-layered. Because $sp^{2}$ hybrid carbon atoms are intensively packed, graphene has a hexagonal lattice structure. Graphene is widely used as the basic building element for carbon materials. These carbon materials have other dimensionalities. In general, these carbon materials include zero-dimensional buckyballs, one-dimensional carbon nanotubes, and three-dimensional bulk graphite materials. Although it takes the form of a laminated sheet which is like thin paper, the single-layer atom thickness is measured at 0.335 nm [19,20]. It is currently the thinnest known two-dimensional material [21]. The wide use of graphene is not separated from its good performance: its theoretical strength remains at 125 GPa [22], which is better than that of steel. More specifically, its strength is 100 times better. Besides this, the elasticity modulus of grapheme reaches 1.0 TPa [23]. Graphene is comparable with carbon nanotubes; its thermal conductivity is 5300 W/(m·K) [24], to which silver and other metallic materials cannot be compared; its electronic mobility reaches $2\times 10^{5}$ cm^2^/(V · s); and its excellent electrical conductivity performance is the best among existing high-temperature superconducting materials [25]. It should also be mentioned that graphene has quantum hall effects and other properties at room temperature [26]. Due to graphene’s excellent electronic and mechanical properties, it is often used in sensors, nanoelectronics, biomedical science, and so on. Recently, Xiang et al. fabricated mechanically robust healable shape memory polymeric films with graphene that can simultaneously heal mechanical damage and the fatigued shape memory function [27]. Huang and colleagues manufactured a composite by adding a low proportion of graphene into the photocrosslinking four-arm poly(ethylene glycol)-poly(epsilon-caprolactone), which showed that graphene had great potential for modifying biosafe shape memory polymers with various excellent performance indicators and broadening its applications in biomedical fields [28]. In addition to these applications, graphene is an ideal filler and a substitute for nanofillers in the fabrication of SMP nanocomposites due to its advantages of high thermal conductivity and great toughness [29]. In this work, we added graphene oxide (GO) into epoxy resin in order to manufacture SMP nanocomposite specimens by adopting an improved preparation process with the aims of highly strengthening its mechanical properties and preserving the shape effects of the SMP. In this study, the thermal, mechanical, and shape memory properties of epoxy polymer composite specimens with different GO contents were studied by adjusting the contents of the enhanced components.

## 2. Experimental

### 2.1. Materials

The sources of the materials that were used in this experiment are as follows. Graphite powder was purchased from Nanjing Kefu Nanomaterials Technology Co., Ltd., Nanjing, China. The matrix used in this paper was epoxy vinyl ester resin (DERAKANE^®^ 411-350 vinyl ester resin), which was produced by Harbin Liyuan Chemical Reagents Co., Ltd., Zhengzhou, China. Methyl ethyl ketone peroxide and dimethylaniline were used as a hardening agent and accelerating agent, respectively, and were produced by Qingdao Hemei Industry and Trade Co., Ltd., Qingdao, China. The weight ratio of the epoxy resin, hardening agent and accelerating agent was 100:1:0.25. Besides these, acetone, potassium permanganate (KMnO_4_), hydrochloric acid (HCl), sodium nitrate (NaNO_3_), sulfuric acid (H_2_SO_4_), and hydrogen peroxide (H_2_O_2_) were purchased from Tangshan Shiqian Industry Co., Ltd., Tangshan, China.

### 2.2. Synthesis of Graphene Oxide (GO)

For the sake of synthesizing GO in an effective way, a modified Hummers’ method was used [30]. In order to achieve the aim of synthesizing GO, 1 g of graphite powder was stirred in 120 mL concentrated H_2_SO_4_ for 2 h. Then, 1 g of NaNO_3_ (over a period of 2 min) and 6 g of KMnO_4_ (over a period of 1 h) were gradually added into the solution. The solution was stirred for 72 h. Then, 600 mL of deionized water was poured into the solution. After stirring the solution for 30 min, H_2_O_2_ was gradually added. The adding of H_2_O_2_ was not stopped until a yellow solution was obtained. Lastly, the mixture was centrifuged at 4000 rpm and sonicated for 2 min. The mixture was also washed with a mixture (1:2 *w*/*w*) of HCl and water.

### 2.3. Fabrication of Epoxy Resin/GO Nanocomposites

Epoxy resin/GO nanocomposites were manufactured according to the following process. Initially, different proportions of GO (0 wt %, 0.4 wt %, 0.8 wt %, and 1.2 wt % relative to epoxy resin) were mixed with a certain amount of acetone (100 mL) and ultrasonicated for 3 h at 30 °C; then, epoxy resin was preheated in a water bath at 60 °C for 15 min. After that, the mixture of GO and acetone was added to the epoxy resin in a water bath at 60 °Cand stirred for 3 h to ensure good dispersion, and the acetone solvent was removed from the mixture solution. Subsequently, the mixture was put into a vacuum drying box for 12 h at 80 °C. After that, the curing agent was preheated in a water bath at 70 °C for 15 min, added to the mixing system, and slightly stirred at 70 °C for 10 min, then put into a vacuum drying box for 10 min at 50 °C. Afterwards, the mixture system was poured into the mold and vacuum treatment was carried out at the same time as curing, followed by curing at room temperature for 12 h. The sample preparation and testing were performed based on the ASTM standard. The dimensions of the samples used in the static tensile test (ASTM D638), three-point bending test (ASTM D7264), low-velocity impact test (ASTM D5420), and shape memory test were 180 × 19 × 3.2 mm^3^, 80 × 13 × 4 mm^3^, 100 × 100 × 3 mm^3^, and 80 × 6 × 2 mm^3^, respectively.

### 2.4. Characterization

In order to characterize the microstructures of nanocomposite samples, scanning electron microscopy (SEM) and transmission electron microscopy (TEM) were adopted. SEM and TEM (FEI Tecnai G2 F20S-TWIN) (JEOL, Tokyo, Japan) were performed with accelerating voltages reaching 200 kV and 30 kV, respectively.

AFM analysis was done in tapping mode by an E-Sweep instrument with a Nanonavi II controller (Seiko instruments, Inc., Tokyo, Japan) to measure the thickness of the GO layers. For this purpose, a much-diluted GO solution in acetone solvent was placed onto freshly cleaved smooth mica substrates to obtain a single layer of GO.

Raman spectra were obtained using a multichannel confocal micro spectrometer which had a laser wavelength of 535 nm.

X-ray diffraction (XRD) analysis was carried out on a diffractometer (Bruker Co., Ltd., Hamburg, Germany) with Cu Kα radiation (λ = 0.154 nm) at an operating voltage of 40 kV and electric current at 40 mA over the range of 5° to 40° and at a scan rate of 3°/min.

X-ray photoelectron spectroscopy (XPS) was carried out using a Kratos Axis Ultra DLD X-ray photoelectron spectroscope (Malvern Instruments, Leeds, UK), and the Al Kα radiation was 1486.6 eV.

Dynamic mechanical performance was studied using a DMA Q800A TA (TA Co., New York, NY, USA), and the storage modulus (E′) and loss tangent (tanδ) versus temperature curves were acquired according to this test. These 3 mm × 10 mm × 40 mm samples were scanned at temperatures ranging from 25 °Cto 125 °C. The heating rate was 3 °C/min. A frequency of 10 Hz and a maximum amplitude of 120 µm were selected as parameters when the dynamic mechanical analysis (DMA) device was used.

The thermogravimetric analysis of the epoxy resin with GO was done on a TGA205-type thermogravimetric analyzer (Perkin-Elmer, San Francisco, CA, USA). In a nitrogen environment with a nitrogen flow rate of 20 mL/min, the heating rate was held at 20 °C/min and the test temperature ranged from 40 °C to 600 °C.

The static mechanical property test was carried out on a universal testing machine (Instron 4505, Bremen, Germany) at a cross-head rate of 2 mm/min. The specimen was stretched to entire failure at a constant rate, and the static tensile performance of the epoxy resin with different GO contents were characterized by engineering stress and engineering strain.

The three point bending tests were done on a Zwich-roell Z010 servo-electri testing machine (STA449, Selbe, German). They were carried out under room temperature. The cross-head rate was 1 mm/min.

Low-velocity impact testing was carried out through the use of a PC-driven Instron Dynatup 9250HV pneumatic-aided drop-weight impact testing machine (NETZSCH Scientific Instruments Trading Ltd., Freiburg, Germany) under room temperature. The machine has a pneumatic brake to prevent strikes. Besides this, the drop height was set in accordance with the impact energy. The initial energy was preset at 30 J in this study.

**Figure 2:**
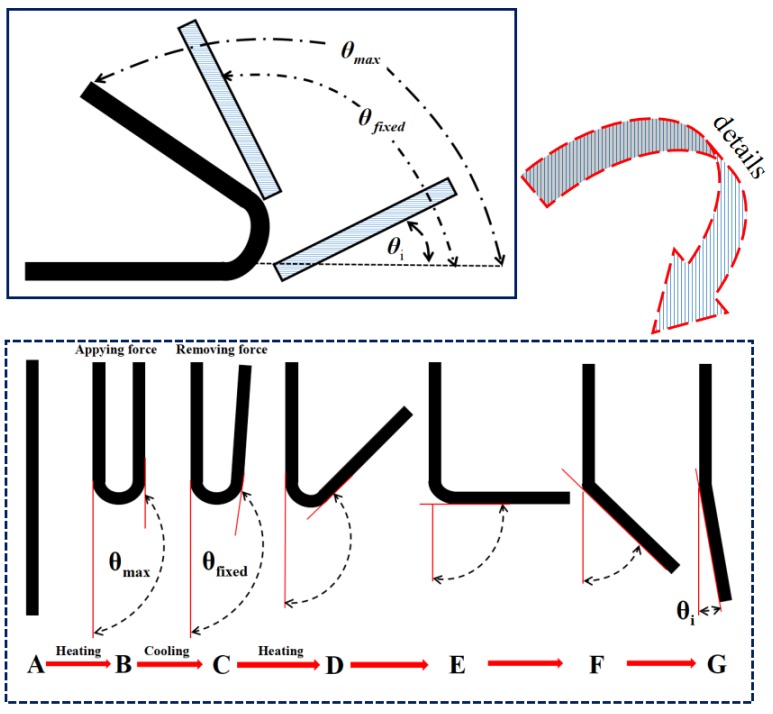
Schematic diagram of the folding deploy shape memory testing.

“Folding deploy shape memory testing” was carried out, which is an experimental method. In order for the specimens to be bent at the largest deformation angle, the specimens were heated above the thermal deformation temperature. The specimens were maintained for some time until their temperature was the same as the room temperature. When the specimen’s shape was preserved, external force was applied, maintained for 2 min, and then released. When the external force was released, there was minor elastic recovery in the specimens, whose angle became $\theta_{fixes}$ after recovery. Later, the specimens were reheated above the thermal deformation temperature at a 5 °C/min rate. The specimen’s bending angle $\theta_{i}$ varied with the rising temperature T during the process. The final bending angle was denoted by $\theta_{final}$. It was found that changes in time *t* and temperature T had a close correspondence to the angle $\theta_{i}$ that was recorded. Figure 2 includes a schematic diagram that presents the shape memory performance test.

(1) $$\mathrm{Shape}\;\mathrm{retention}\;\mathrm{ratio}:\;R_{f}=\frac{\theta_{fixed}}{\theta_{max}}\times 100\%$$

(2) $$\mathrm{Shape}\;\mathrm{recovery}\;\mathrm{ratio}:\;R_{r}=\frac{\theta_{max}-\theta_{i}}{\theta_{max}}\times 100\%$$

(3) $$\mathrm{Shape}\;\mathrm{recovery}\;\mathrm{rate}:\;V_{r}=\frac{\pi \left(\theta_{fixed\;}-\theta_{i}\right)}{180t}$$

## 3. Results and Discussion

### 3.1. Characterizations of Microstructure

**Figure 3:**
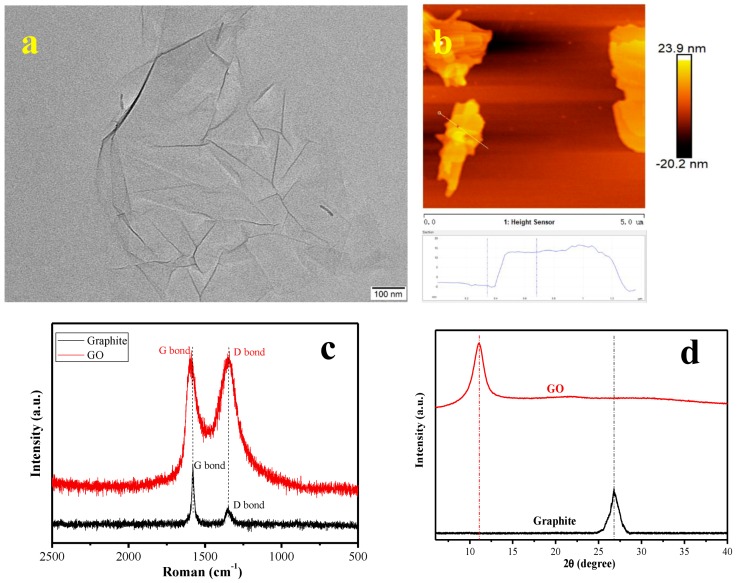
(**a**) TEM image of graphene oxide (GO), (**b**) AFM image of GO, (**c**) the Raman spectra of graphite and GO, and (**d**) XRD pattern of graphite and GO.

GO’s morphologies were characterized by TEM and AFM, as shown in Figure 3a,b. According to Figure 3a, the exfoliated GO sheets had a transparent clean structure and showed few thin ripples. In addition to the sheet-like morphology consistent with the TEM characterization which can be seen in Figure 3b, the AFM analysis showed that GO’s thickness in this work was 12.31 nm on average. Figure 3c shows the Raman spectra of graphite and GO. ID/IG, which refers to the ratio of the G bond’s intensity to the D bond’s intensity, is often used to indicate a carbonaceous material’s regularity. In the Raman spectrum of the graphite sample, there is a distinct G bond at 1560 cm^−1^, and a minor protrusion (D bond) is found around 1310 cm^−1^. The GO sample’s Raman spectrum shows a G band and a D band at ca. 1581 cm^−1^ and 1313 cm^−1^, respectively. It was found that the G bands for graphite and GO were red-shifted from 1560 cm^−1^ to ca. 1581 cm^−1^, which means that an oxidation reaction happened when the graphite was being stripped. What is equally important is that when GO was being prepared, the ID/IG ratio obviously increased. This means that graphite’s regularity was seriously damaged when the graphite was oxidized, which further reflects that the graphite was stripped into GO. Figure 3d presents the XRD patterns of graphite and GO. According to Figure 3d, graphite shows an obvious characteristic peak at θ = 26.8°, corresponding to the (002) crystal plane of the graphite structure. It is noteworthy that there is no characteristic peak for GO at this point, but it has a characteristic diffraction peak at θ = 10.9°, which corresponds to the (100) crystal plane of GO. Compared with graphite, the characteristic diffraction peak of GO shifted to a smaller angle, which indicates that lattice distortion of the graphene oxide carbon structure occurred. It further indicates that a large number of functional groups merged into the interlayer of graphite during the oxidation process and thus changed the intrinsic structure of the graphite.

**Figure 4:**
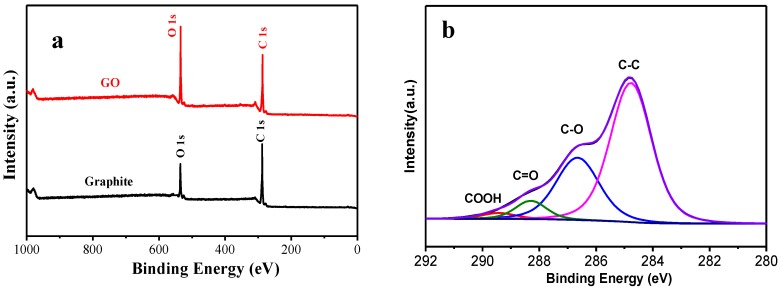

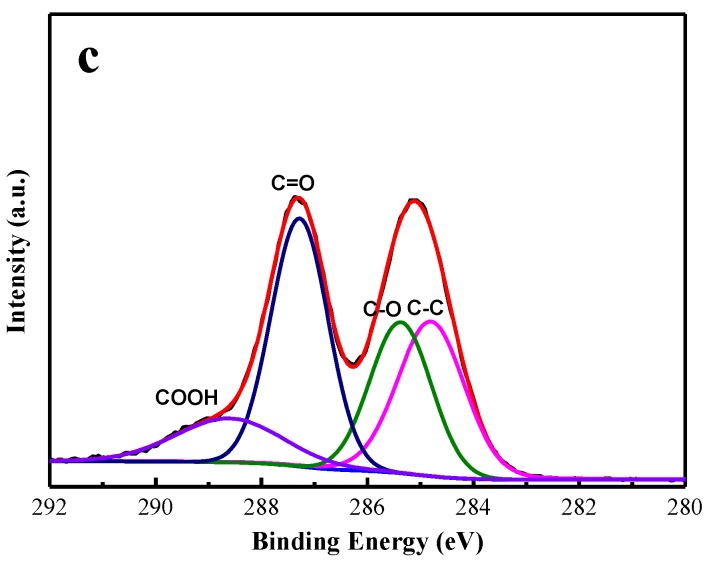
(**a**) XPS survey scans of graphite and GO; C1s XPS spectra of (**b**) graphite and (**c**) GO.

XPS was used to show the characteristics of the chemical compositions of graphite and GO, which are shown in Figure 4. Figure 4a is a survey scan of XPS of the samples. From these, it can be seen that in all of the test samples, C1s and O1s peaks were obvious. The C1s XPS spectra of graphite and GO are shown in Figure 4b,c. It is clearly shown that carbon atoms exist in different functional groups. In terms of graphite’s C1s XPS spectrum, four bands of graphite are observed at about 284.6 eV, 286.8 eV, 288.2 eV, and 289.6 eV, which correspond to C=C bonds, C–O bonds, C=O bonds, and O–C=O bonds, respectively. It should be mentioned that all of the C1s peak areas in C–O, C=O, and O–C=O of the GO sample increased in comparison with those of graphite. The main reason for this is that oxygen-containing functional groups were introduced to the GO surface. GO’s C1s XPS spectrum shows the success of the intercalation oxidation reaction of graphite. Hummers’ method proved applicable in this study since there was an obvious decrease in the carbon/oxygen ratio.

### 3.2. Thermal Properties

**Figure 5:**
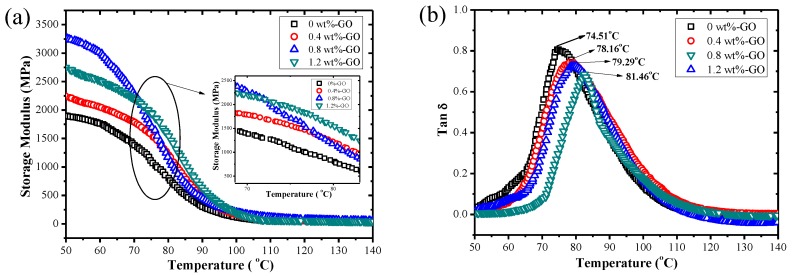
DMA curves of storage modulus (**a**) and tan delta (**b**) versus temperature for neat epoxy resin and GO-toughened epoxy resin composites.

Figure 5 includes DMA curves of the storage modulus (E′) and tan delta (tan δ) versus temperature curves for neat epoxy resin and GO-toughened epoxy resin composites. Figure 5a indicates that the storage modulus of GO-toughened epoxy resin with different mass fractions were evidently increased when GO was added at different temperatures. When the GO contents were 0.4 wt % and 0.8 wt %, the storage moduli of the epoxy resin polymer composites increased with the increase in GO content. It also heightened the interfacial stress transfer and raised the stiffness of the epoxy resin polymer composites. It is noteworthy that when the GO content was 1.2 wt %, the storage modulus of GO toughened epoxy resin composite was reduced. This is probably because when the GO content rises to a certain critical value, the dispersion of GO in the epoxy resin worsens. As a result, the stress transfer effect was weakened, which resulted in a decrease in the epoxy resin polymer composite’s storage modulus.

Figure 5b shows that the glass transition temperature ($T_{g}$) of pure epoxy system reaches 74.51 °C and the $T_{g}$ of polymer composite after GO filling is increased. As the GO content increased, $T_{g}$ moved towards a higher temperature. The reason could be that the polymer composite was highly cross-linked, which made it have a stronger acting force to remain unchanged. When the GO content was increased, the cross-linking structure was more compact, and the restriction degree of chain motion increased. To achieve the chain motion, test samples that were cross-linked required more free space as well as more energy. Therefore, a higher temperature was needed to obtain the energy for the chain motion. However, only when there was a higher temperature, would the inside of the SMP nanocomposite have larger free space as well as more energy to realize the motion of the molecular chain segments, thus causing the increase of $T_{g}$. Furthermore, we paid attention to the polymer composite’s shape memory performance when $T_{g}$ was increased. Shape memory performance is mainly affected by the cross-linking structure of the composite. The cross-linked composite’s recovery force is stronger than that of the polymer matrix, which results in the need for more energy (higher temperature) to achieve shape recovery for composites with more cross-linked structures units [31]. Furthermore, the loss factors of the epoxy resin polymer composites were reduced when the GO content was increased and were lower than the neat epoxy resin’s tan delta values.

**Figure 6:**
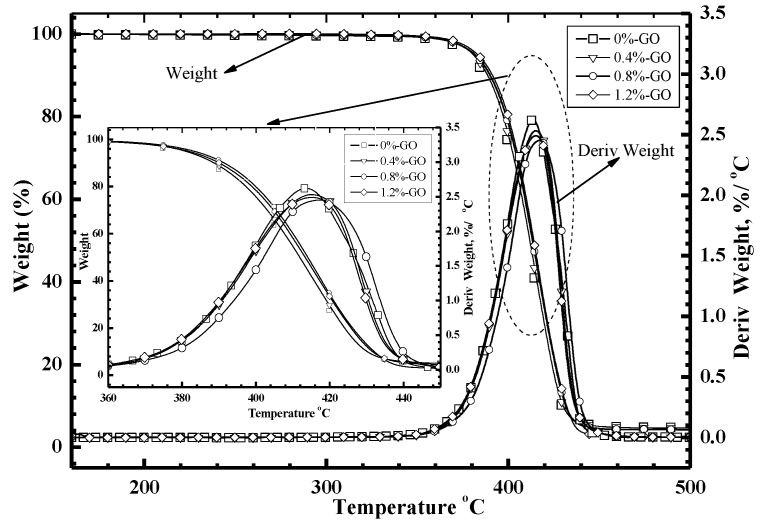
The TGA and DTG curves of neat epoxy resin and nanocomposite samples.

Figure 6 shows the TGA and DTG curves of epoxy resin into which different GO contents were added. As shown in Figure 6, the composites begin decreasing in weight when the temperature reached 360 °C. The relationship between temperature and weight in the polymer composites that contained GO was not different from that of the pure epoxy system. With the augmentation in GO mass fraction, the critical temperature when the composite started being decomposed increased as a whole. When the epoxy resin polymer composites’ mass loss was 5%, the corresponding temperatures of the four systems of composites with the GO content of 0 wt %, 0.4 wt %, 0.8 wt % and 1.2 wt % were 374.36 °C, 374.61 °C, 376.23 °C and 375.67 °C, respectively. This is mainly because GO forms a network structure in the sample, which will limit the spilling of micro-molecules that are formed during the process of decomposition. Because of that, the decomposition temperature of composite is increased. In terms of the rate of weight loss of sample that is shown in the DTG curve, the peak value means the sample’s highest thermal decomposition rate. It should be mentioned that the temperature that corresponds to the peak value is the highest decomposition temperature. From the DTG curve, we can see that the pure epoxy system’s maximum decomposition temperature was the lowest, and the thermal decomposition rate was the highest. When the GO mass fraction was controlled within a certain range, the composite’s maximum thermal decomposition temperature increased, and the thermal decomposition rate decreased with the augmentation in the mass fraction of GO. When the GO mass fraction was over 0.8 wt %, the maximum thermal decomposition temperature took the tendency of a reduction with the augment in the GO mass fraction. Besides, the thermal decomposition rate increased accordingly. When the content of GO contained in the epoxy resin matrix was 0.8 wt %, the GO-reinforced epoxy resin polymer composite system had the best thermal properties.

### 3.3. Mechanical Properties

**Figure 7:**
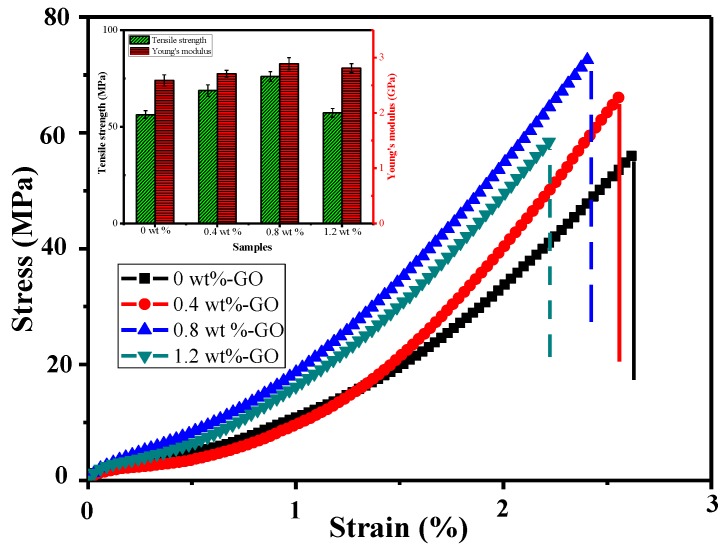
Stress-strain curves of the GO-toughened epoxy resin.

The tensile performance of the epoxy resin polymer composites was evaluated in a uniaxial tensile test. Stress–strain curves of the GO-toughened epoxy resins are presented in Figure 7. Furthermore, tensile mechanical parameters of nanocomposites with various of GO content are shown in Table 1. The fracture strains of specimens with GO contents of 0 wt %, 0.4 wt %, 0.8 wt %, and 1.2 wt % were all more than 2%. When the GO content was greater than 0.8 wt %, it can be seen clearly that the strain value of the sample remarkably declined. All samples had uniform elongation, with the exception of the sample with a GO content of 1.2 wt %. The reason for this is that when the dosage of GO is too high, it is harder for the GO in the epoxy resin matrix to disperse. The failure of the samples basically resulted from superfluous GO agglomeration. It can be observed that all the epoxy resin polymer composite specimens mixed with GO showed higher tensile strength than the neat epoxy resin without GO. The fracture stresses all exceeded 50 MPa in the systems with GO contents of 0 wt %, 0.4 wt %, 0.8 wt %, and 1.2 wt %. However, it is noteworthy that when the mass fraction of GO was above 0.8 wt %, the fracture stress exhibited a downward tendency. The greatest fracture stress was found in the sample with a GO content of 0.8 wt %, and it was 34.9% greater than that of the neat epoxy resin. The maximum fracture stresses of samples with GO contents of 0.4 wt % and 1.2 wt % were greater than the maximum fracture stress of the neat epoxy resin by 22.1% and 3.3%, respectively.

**Figure 8:**
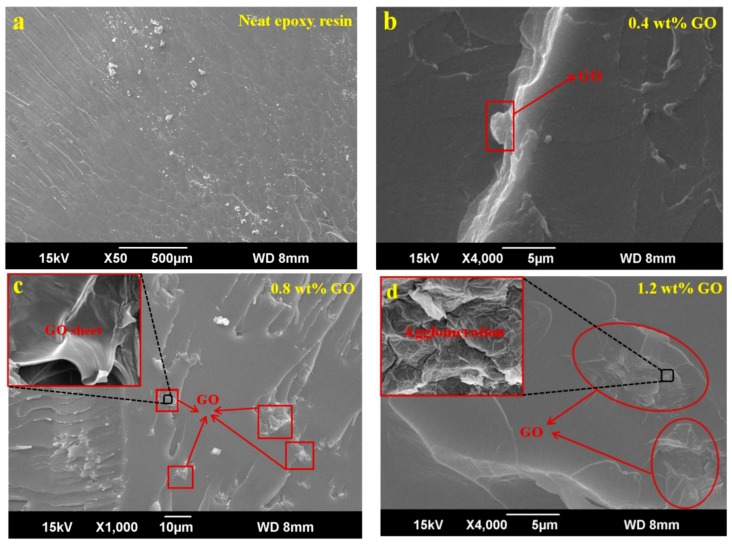
The SEM images of tensile fracture sections of GO/epoxy resin composites with different GO content: (**a**) neat epoxy resin, (**b**) 0.4 wt %, (**c**) 0.8 wt % and (**d**) 1.2 wt %.

Figure 8 presents SEM images of the GO/epoxy resin composites. It can be seen in the figure that the tensile fracture cross-section of the pure epoxy resin is flatter. When a small amount of GO (0.4 wt %) was introduced, pleated unevenness appeared in the sample’s tensile fracture sections. The GO sheets that were fractured were found to be distributed in a uniform manner, which means that GO could be evenly dispersed in the matrix. Furthermore, as seen from Figure 8c, when the content of GO was 0.8 wt %, the aggregation phenomenon was not obvious; however, when the GO mass fraction was increased to 1.2 wt %, there was evident reuniting in the epoxy resin, and the multilayer GO was stacked together which is clearly observed in the Figure 8d. When the GO content increased, the composites’ tensile fracture section tended to be increasingly rough and an obvious tough fracture morphology was observed. We also found that the roughness of the sample containing 0.8 wt % GO was the greatest. In the fracture section of the sample with a 0.8 wt % GO content, an obvious layered fracture can be seen, which reflects that the filler network and matrix actively interacted. The reason is that GO containing a large number of hydrophilic groups can be more uniformly dispersed in epoxy resin. Furthermore, the GO surface contains a large number of active groups. Active groups can achieve strong interaction with epoxy resin by covalent bonding or hydrogen bonding, which greatly improves the interfacial compatibility of the GO and epoxy matrix. As GO content was added in a continuous way, the sample’s tensile fracture section was less rough compared with that of the 0.8 wt % sample. The reason is that when the amount of GO is excessive, the dispersion of GO in polymer matrix worsens, which causes a certain degree of agglomeration. Tensile fracture of the sample with 1.2 wt % GO content has a tendency towards brittle fracture, thus causing roughness of epoxy resin containing 1.2 wt % GO decreased in SEM images compared with sample with 0.8 wt % GO.

**Figure 9:**
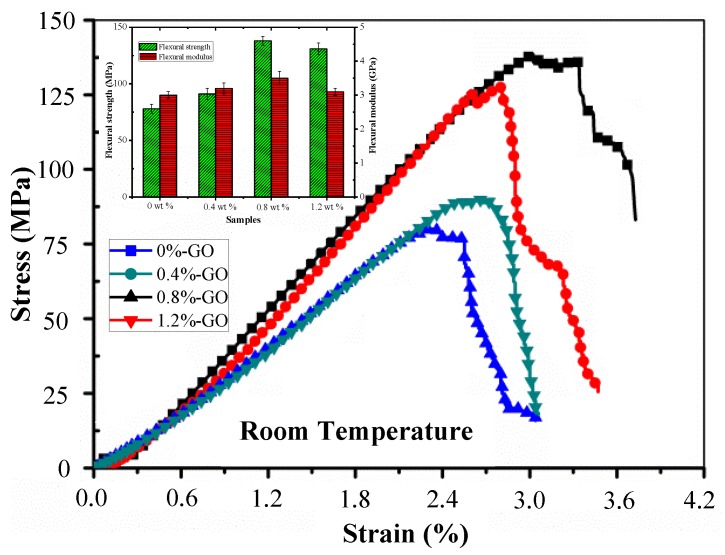
Flexural stress-strain curves of epoxy resin composite samples with different GO mass fractions at room temperature.

The flexural stress–strain curves of the epoxy resin composite samples with various GO mass fractions under room temperature conditions are presented in Figure 9. According to the figure, the composite specimens with different contents of fillers show the same tendency: as the stress is enhanced, the linear strain increases until the specimens are damaged suddenly. The flexural strengths, strains and moduli of the epoxy resin polymer composites with various GO contents measured from specimens in room temperature conditions are shown in Table 2. At room temperature, it was found that the composite samples with GO contents of 0 wt %, 0.4 wt %, 0.8 wt %, and 1.2 wt % showed the flexural strengths of 78.13 MPa, 91.57 MPa, 139.06 MPa, and 131.49 MPa, respectively, and moduli of 3.06 GPa, 3.23 GPa, 3.51 GPa, and 3.47 GPa, respectively. As the GO content increased, the flexural properties of the composite were improved to a certain degree. For the specimens with GO mass fractions of 0.4 wt %, 0.8 wt %, and 1.2 wt %, the flexural strengths were greater than that of the neat epoxy resin specimen by 17.20%, 60.93%, and 53.36%, respectively, when tested in room temperature; the moduli were also greater by 5.56%, 14.71%, and 13.39%, respectively. The sample with a GO content of 0.8 wt % had the best flexural strength and modulus among all the bending samples. When the GO content was greater than 0.8 wt %, the flexural properties of the sample declined. The main reason for this is the inhomogeneity of the epoxy resin cured product network leading to the formation of a micro nonuniform continuous structure. This structure resulted in a decrease in the strength of the material. Thus, it can be seen that the addition of GO in the epoxy resin can remarkably increase the flexural strength. Although the samples with different GO contents that were used in the static tensile testing and the three-point bending testing were analogous, it was found that in the three-point bending tests, GO’s enhancement effect was more obvious.

**Figure 10:**
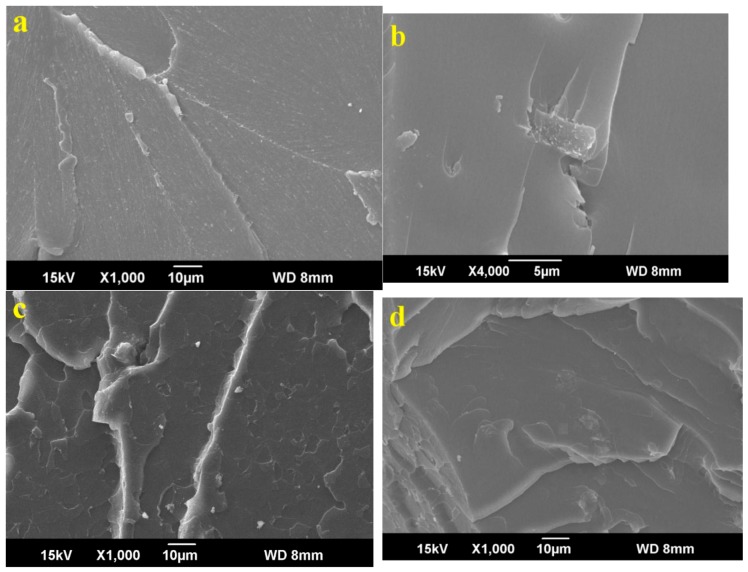
The SEM images of GO/epoxy resin composites with different GO contents of: (**a**) 0 wt %, (**b**) 0.4 wt %, (**c**) 0.8 wt % and (**d**) 1.2 wt % in flexural tests.

Figure 10 presents the SEM images of GO/epoxy resin samples with various GO mass fractions in flexural tests. According to Figure 10, when the GO content was increased, the cross section became increasingly rough at the very beginning, but the roughness then weakened. For the pure epoxy resin containing no GO and the nanocomposites with low GO content (0.4 wt %), the samples’ fracture sections were flat, which shows that the bending fracture was a typical brittle fracture. When the GO content was increased, the composites’ fracture section was increasingly rough, and a more obvious tough fracture morphology was observed. The roughness of the nanocomposite with 0.8 wt % GO mass fraction was the maximum. It is worth mentioning that the fracture section of the specimen containing 0.8 wt % GO takes the form of a ladder, and the layered fracture is distinct, which indicates that GO and epoxy resin have more active interaction. When the GO content was gradually increased, the roughness of the fracture sections of samples decreased compared with that of the 0.8 wt % specimen.

**Figure 11:**
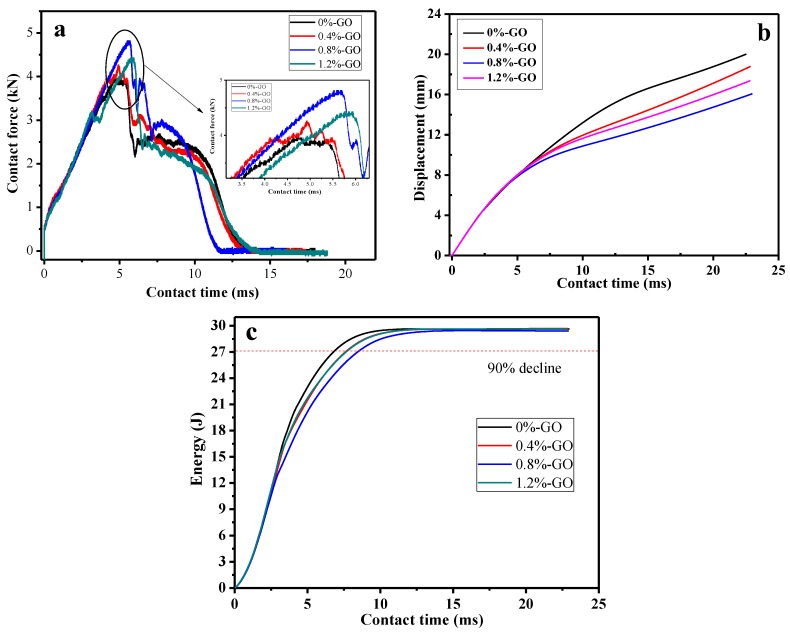
Typical graphs of epoxy resin polymer composite specimens with different GO contents after impact: (**a**) contact force-time (F-t) curves, (**b**) displacement-time (D-t) curves and (**c**) energy-time (E-t) curves.

Figure 11 shows typical graphs of epoxy polymer composite specimens with different GO contents impacted at low velocity with energy of 30 J. It was found that contact force is a significant parameter in the analysis of low-velocity impact because it is a reaction force of the impactor on the sample. It was found that the force versus time curves of the composite specimens took the shape of a mountain, as shown in Figure 11a. It can be seen that the F-t curves of the composite specimen showed the tendency of sharply rising at first and then declining. When the GO content was 0.8%, the impact strength was the greatest—21.6% greater than that of the neat epoxy resin specimen. With an increase of the GO content, it is noteworthy that the impact strength of composite specimens declined. Figure 11b shows that the deformation versus time curves of the composite specimens and the maximum displacement are necessary parameters in the analysis of low-velocity impacts. The sample with no GO was deformed more obviously than the samples with GO mass fractions of 0.4 wt %, 0.8 wt %, and 1.2 wt %. Besides this, among all the specimens mixed with GO, specimens with GO mass fractions of 0.8 wt % had the minimum deformation. In addition, energy functioned as a significant parameter in the evaluation of the damage caused by low-velocity impacts on epoxy polymer composites. The E-t curves of the composite specimens are shown in Figure 11c. According to Figure 11c, the energy of specimens that had no GO was the highest. However, specimens with GO mass fractions of 0.8 wt % had the lowest energy. On the basis of the data drawn from the low-velocity impact experiment, we reached the conclusion that composite specimens can obtain more elastic deformation energy by means of adding GO.

**Figure 12:**
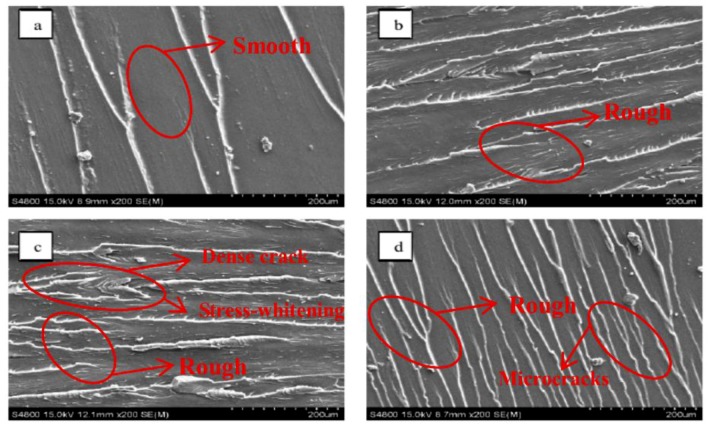
SEM images of the impacted fracture surfaces of SMEP specimens with different GO contents of (**a**) 0 wt %, (**b**) 0.4 wt %, (**c**) 0.8 wt % and (**d**) 1.2 wt % after impact test.

Figure 12 presents SEM images of the impacted fracture surfaces of specimens with GO mass fractions of 0 wt %, 0.4 wt %, 0.8 wt %, and 1.2 wt %. As shown in Figure 12a, the overall fracture surface morphology of the neat epoxy resin was relatively smooth, with small unidirectional crack branches propagating linearly. This indicates that the cracks were not subjected to any obstruction during the expansion. They were typically brittle fractures. In contrast, in Figure 12b–d, the fracture surfaces were rough due to the addition of GO, with dense crack branches. The cracks showed obvious deflection and disproportionation, resulting in a large number of microcracks. It is also observable that these microcracks were suppressed and arrested during the process of development, showing the transition to ductile fracture. In particular, in the specimen with a GO mass fraction of 0.8 wt %, a large number of cracks were produced in the curing system, which were caused by low-velocity impact. The fracture surface was feathery and coarse and was surrounded by significant stress-whitening. Clearly, the addition of GO is conducive to improving the toughness of the resin curing system. Its toughening mechanism may result from the heterogeneity of the cured cross-linked network, thereby forming a microscopic heterogeneous continuous two-phase structure to achieve energy dissipation.

### 3.4. Shape Memory Properties

**Figure 13:**
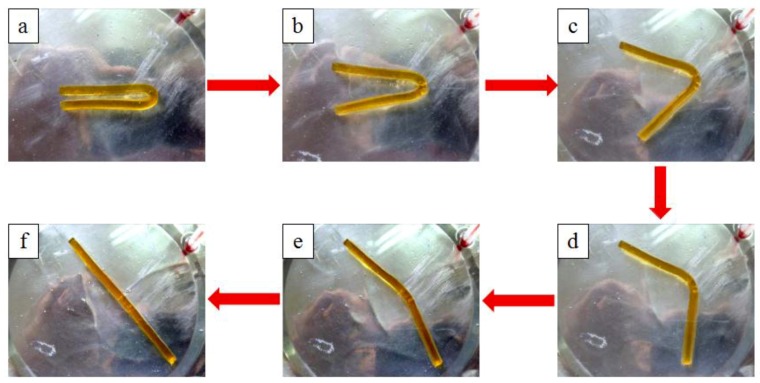
Process for the test of shape memory properties.

Figure 13 demonstrates images from the testing of the shape memory performance of the epoxy polymer composite specimens. The test was mainly used to test their thermodynamic deformation.

**Figure 14:**
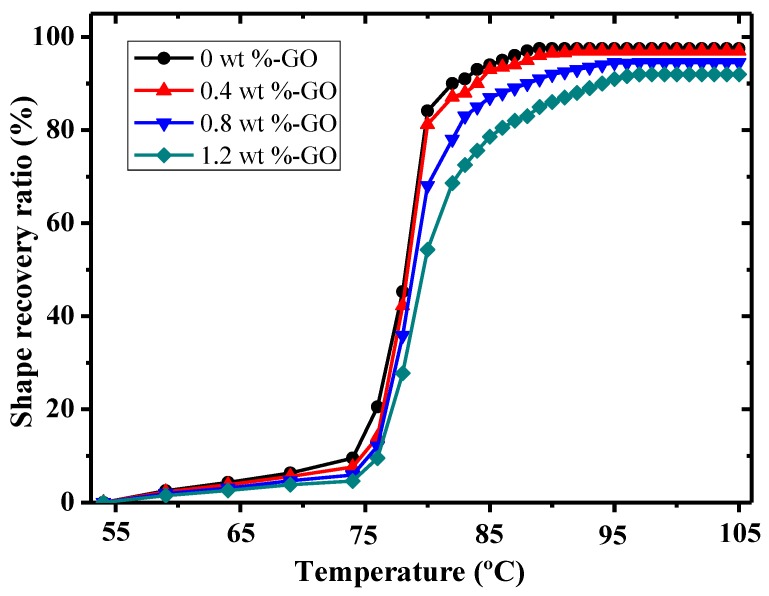
The shape recovery ratio of SMEP composites with different GO contents at different temperatures.

Figure 14 shows the shape recovery ratio vs. temperature curves of shape memory epoxy polymer composite specimens with varying GO contents. All specimens had a parallel tendency for the shape recovery ratio to increase in a monotonic and sharp manner with rising temperature, but they eventually tended to be stable. Furthermore, when the temperature reached 74 °C, the shape recovery ratios of samples with GO contents of 0 wt %, 0.4 wt %, 0.8 wt %, and 1.2 wt % were 11.8%, 9.3%, 7.8%, and 6.1%, respectively. When the temperature was greater than $T_{g}$, all samples’ shape recovery ratios improved quickly with the continual increase of temperature, which may be accounted for in this way: structures that are closely cross-linked exert strong constrained force on the molecular chains. Therefore, the molecular chains of this kind of specimen need the greater free space and energy that are gained when there is higher temperature so as to achieve shape recovery. The shape recovery ratio of the neat epoxy resin sample was greater than those of the other samples, and all samples’ shape recovery ratios were improved by more than 90%. With the increase of filler content, the addition of fillers will restrict the activity of polymer molecular chains to a certain extent, resulting in the increase of resistance in the recovery process of composites and the decrease of the shape recovery ratio [32,33]. As a result, the addition of fillers has no obvious improvement for the the shape recovery performance of the matrix; however, it can be seen from the shape memory test that the overall harmful impact of filler addition on shape memory performance of composite is very minor. All of these show that the epoxy polymer composite’s shape recovery performance was good.

**Figure 15:**
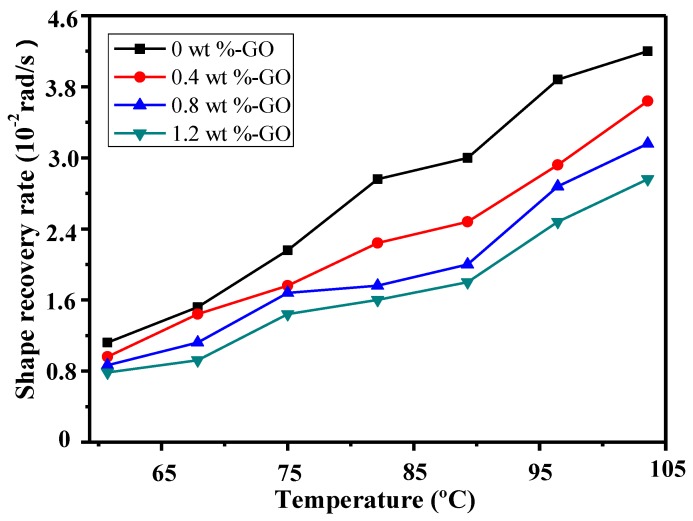
The shape recovery rate of SMEP composites with different GO contents at different temperatures.

Figure 15 presents the shape recovery rates of epoxy polymer composite samples containing varying GO mass fractions under different temperatures. It can be seen that all samples’ shape recovery rates rose when the temperature rose. Furthermore, as the GO mass fraction rose, the shape recovery rate of the sample declined. The reason for this is that the recovery force of the composite structure in a polymer composite exceeds that in a polymer matrix. A sample that has more composite structures requires more energy to accomplish shape recovery. If temperature is invariable, in order to achieve shape memory recovery, the sample unavoidably requires more time. As a result, the shape recovery rate is reduced.

## 4. Conclusions

In this paper, the thermal, mechanical, and shape memory performance of epoxy resin composite specimens with different GO mass fractions were analyzed by DMA, TGA, static tensile, three-point bending, impact, and shape memory tests. According to the experimental results herein, when the GO content was 0.8 wt %, the composite had good shape memory performance and optimal thermal and mechanical performance. This indicates that 0.8 wt % GO content could be the optimum value for an epoxy polymer composite in the proposed manufacturing process. The main conclusions of this study may be summarized as follows:

(1) The *T_g_* of a specimen after GO filling was enhanced, and presented an upward trend with augmenting of the GO content. The maximum thermal decomposition temperature showed a tendency of first increasing and then decreasing with the augmenting of the GO mass fraction. Accordingly, the thermal decomposition rate showed a similar trend.
(2) The tensile fracture stresses and flexural fracture stresses of samples with 0.8 wt % GO content reached their maximum values, which were 34.9% and 60.93% greater than those of neat epoxy resin, respectively.
(3) The greatest impact strength, minimum impact deformation and maximum absorbed energy were obtained in the fabricated samples with 0.8 wt % GO content.
(4) The shape recovery ratio of composites decreased with the increase of GO content, and the shape restoring rate increased as the temperature rose.

## Figures and Tables

**Table 1:** Tensile parameters of nanocomposites with various GO content.

GO Content (wt %)	Fracture Stress (MPa)	Fracture Strain (%)	Young’s Modulus (GPa)
0.0	56.26	2.63	2.59
0.4	68.71	2.57	2.71
0.8	75.93	2.42	2.89
1.2	58.12	2.24	2.81

**Table 2:** Flexural properties of nanocomposites with different GO content at room temperature.

GO Content (wt %)	Flexural Stress (MPa)	Flexural Strain (%)	Flexural Modulus (GPa)
0.0	78.13	2.56	3.06
0.4	91.57	2.69	3.23
0.8	139.06	3.32	3.51
1.2	131.49	2.75	3.47
